# Per- and polyfluoroalkyl substances (PFAS) exposure in melanoma patients: a retrospective study on prognosis and histological features

**DOI:** 10.1186/s12940-022-00944-x

**Published:** 2022-12-09

**Authors:** Paolo Del Fiore, Francesco Cavallin, Marcodomenico Mazza, Clara Benna, Alessandro Dal Monico, Giulia Tadiotto, Irene Russo, Beatrice Ferrazzi, Saveria Tropea, Alessandra Buja, Claudia Cozzolino, Rocco Cappellesso, Lorenzo Nicolè, Luisa Piccin, Jacopo Pigozzo, Vanna Chiarion-Sileni, Antonella Vecchiato, Chiara Menin, Franco Bassetto, Angelo Paolo Dei Tos, Mauro Alaibac, Simone Mocellin

**Affiliations:** 1 Soft-Tissue, Peritoneum and Melanoma Surgical Oncology Unit, Veneto Institute of Oncology IOV-IRCCS, 35128 Padua, Italy; 2 Independent Statistician, 36020 Padua, Solagna Italy; 3 Department of Surgery, Oncology and Gastroenterology (DISCOG), University of Padova, 35128 Padua, Italy; 4 Division of Dermatology, Department of Medicine (DIMED), University of Padova, 35128 Padua, Italy; 5 Postgraduate School of Occupational Medicine, University of Verona, 37129 Verona, Italy; 6 Department of Cardiological, Thoracic, Vascular Sciences and Public Health, University of Padova, 35128 Padua, Italy; 7 Pathological Anatomy Unit, University Hospital of Padova, 35128 Padua, Italy; 8 Department of Medicine (DIMED), Unit of Pathology & Cytopathology, University of Padova, 35128 Padua, Italy; 9 Unit of Surgical Pathology & Cytopathology, Ospedale Dell’Angelo, 30174 Mestre, Italy; 10 Melanoma Unit, Oncology 2, Veneto Institute of Oncology IOV-IRCCS, 35128 Padua, Italy; 11 Immunology and Diagnostic Molecular Oncology Unit, Veneto Institute of Oncology IOV-IRCCS, 35128 Padua, Italy; 12 Clinic of Plastic Surgery, Department of Neuroscience, Padua University Hospital, University of Padova, Padua, Italy; 13 Department of Medicine- DIMED, University of Padova, 35128 Padua, Italy

**Keywords:** PFAS, Perfluoroalkyl substances, Compounds, Melanoma, Cutaneous melanoma, Skin cancer, Endocrine disruptor, Vitamin D, PFOA, PFOS

## Abstract

Per- and polyfluoroalkyl substances (PFAS) are endocrine disrupting chemicals which could be associated with cancer development, such as kidney and testicular cancers, pancreatic and hepatocellular carcinoma and thyroid tumor. Available scientific literature offers no information on the role of PFAS in melanoma development/progression. Since 1965, a massive environmental contamination by PFAS has occurred in northeastern Italy. This study compared histopathology and prognosis between melanoma patients exposed (*n* = 194) and unexposed (*n* = 488) to PFAS. All patients were diagnosed and/or treated for melanoma at the Veneto Oncological Institute and the University Hospital of Padua (Italy) in 1998–2014. Patients were categorized in exposed or unexposed groups according to their home address and the geographical classification of municipalities affected by PFAS contamination as provided by Veneto Government in 2018. Presence of mitoses was found in 70.5% of exposed patients and 58.7% of unexposed patients (*p* = 0.005). Median follow-up was 90 months (IQR 59–136). 5-year overall survival was 83.7% in exposed patients and 88.0% in unexposed patients (*p* = 0.20); 5-year disease-specific survival was 88.0% in exposed patients and 90.9% in unexposed patients (*p* = 0.50); 5-year disease-free survival was 83.8% in exposed patients and 87.3% in unexposed patients (*p* = 0.20). Adjusting for imbalanced characteristics at baseline (presence of mitoses), survival was not statistically different between exposed and unexposed patients (overall survival: HR 1.10, 95% CI 0.77 to 1.58, *p* = 0.57; disease-specific survival: HR 0.99, 95% CI 0.62 to 1.59, *p* = 0.99; disease-free survival: HR 1.10, 95% CI 0.74 to 1.64, *p* = 0.62). Although the magnitude of PFAS exposure was not quantifiable, our findings suggested that exposure to PFAS was associated with higher level of mitosis in melanoma patients, but this did not translate into a survival difference. Further studies are required to investigate this relationship and all effects of PFAS on prognosis.

## Background

**Fig. 1:**
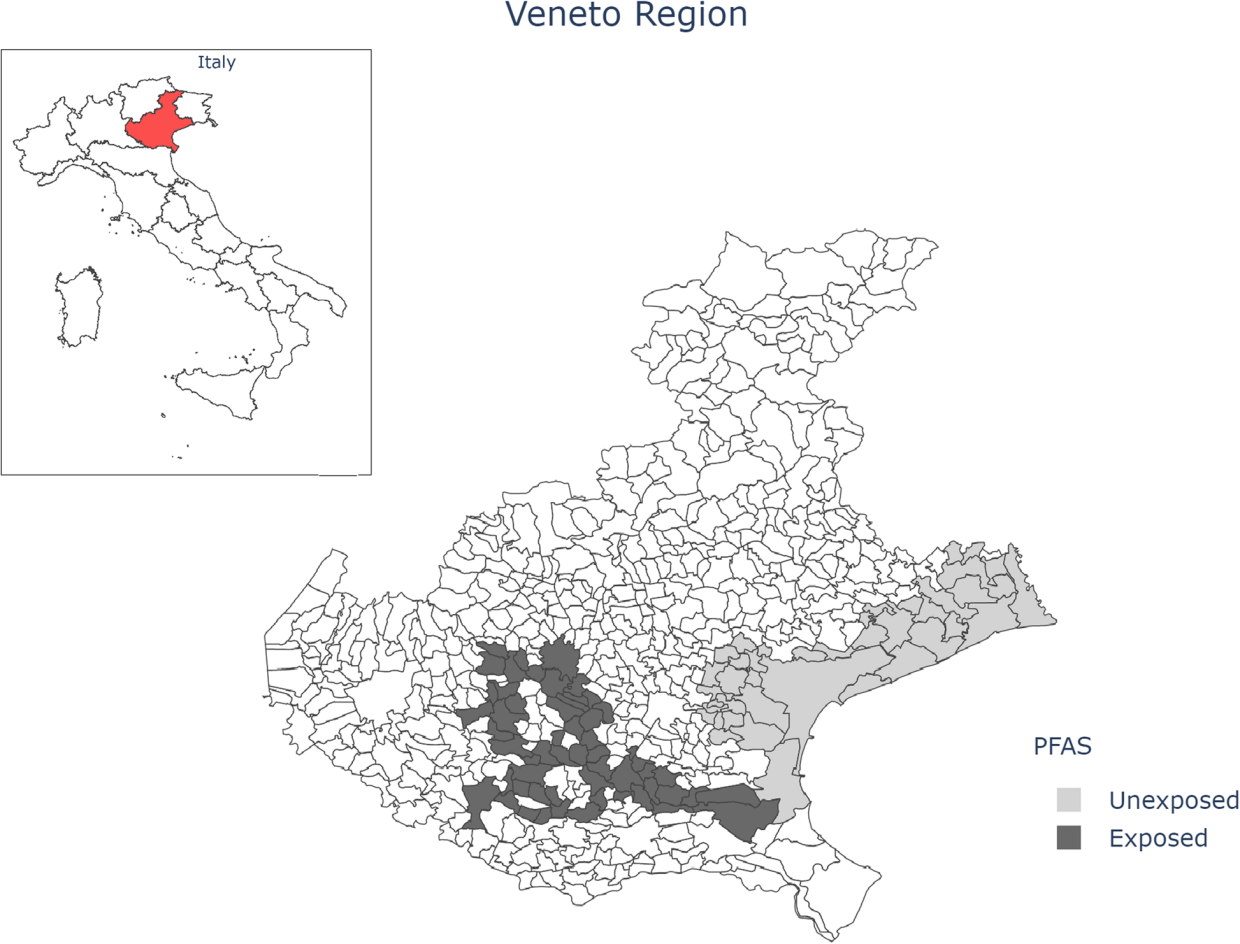
Unexposed and exposed area in Veneto Region

The incidence of melanoma is continuously increasing in both adult and pediatric populations around the world, with a faster pace compared to other malignancy [1, 2]. The development of melanoma is multifactorial and mainly related to ultraviolet light exposure and genetic susceptibility [3]. However, several studies reveal significant correlations between chemical exposure and melanoma incidence [4]. Per-and polyfluoroalkyl substances (PFAS) are a group of man-made organic chemicals that are persistent environmental contaminants because of their resistance to biodegradation, photo-oxidation, direct photolysis, and hydrolysis [5]. PFAS have been manufactured since the 1940s and widely used in a variety of consumer and industrial products (such as carpeting, clothing, upholstery, food paper wrappings, fire-fighting foams) and in processes such as polytetrafluoroethylene (PTFE) polymer production and metal plating. PFAS are persistent and ubiquitously distributed in the environment thus growing into a global contamination problem. Previous studies have associated PFAS with several health conditions such as hepatotoxicity, dyslipidemia, endocrine outcomes, immunotoxicity outcomes, hyperuricemia, pregnancy-induced hypertension and cancer development such as kidney and testicular cancers, pancreatic and hepatocellular carcinoma, and thyroid tumor [6–8]. Cancer is one of the health effects of interest in relation to PFAS exposure [8]. In 2013, the Italian National Research Center (IRSA-CNR), triggered by the outcomes of the European PERFORCE project, found a high presence of PFAS in water and soil of large areas of North-eastern Italy. The main source of contamination was a chemical plant in the Province of Vicenza (Veneto Region), which had produced PFAS compounds since early 60 s. The contaminated area includes 30 small towns of the province of Vicenza, Verona and Padova for a total of approximately 140,000 people (Fig. 1) directly exposed to the PFAS pollution [9, 10]. The “Veneto Cancer Registry” records melanoma as the most common cancer diagnosis in males, the third most common cancer in females under 50 years of age and the sixth most common cancer overall in the Veneto Region (Italy). Based on its endocrine disrupting action and the relationship with vitamin D and mitotic rate [11], we hypothesized that PFAS exposure might affect the biological aggressiveness of melanoma. Hence, we compared melanoma patients stratified by the potential exposure to PFAS, to investigate differences in terms of tumor histological characteristics and prognosis between patients exposed and those unexposed to PFAS.

## Methods

This was a retrospective cohort study on PFAS exposure in patients who were diagnosed and /or treated for primary melanoma at Veneto Institute of Oncology and at the University Hospital of Padua between 1998 and 2014.

### Patients

All patients who were diagnosed and/or treated for primary melanoma in 1998–2014 at Veneto Institute of Oncology and at the University Hospital of Padua (Italy) were considered for inclusion in this study. The two participating hospitals are level III referral centers located in the Veneto Region (North-eastern Italy). Most patients are referred for diagnosis and/or first-line treatment, while some patients are referred for disease progression after being treated in local level II centers. Main inclusion criteria were age ≥ 15 years and living in the Veneto Region. In 2018, the regional administration produced a document (no. 691 of 21 May 2018) where regional areas were classified according to PFAS contamination [12]. In our study, patients living in PFAS areas until the initial diagnosis of melanoma were included in the exposed cohort, while patients living outside PFAS areas in the province of Venice were included in the unexposed cohort. Patients did not report a change in home address. In the exposed cohort, the source of water was either municipal systems or private wells. The inclusion period (1998–2014) was chosen to potentially achieve a minimum follow-up period of 5 years at the analysis.

### Diagnosis and treatment

All melanoma diagnoses were histologically confirmed according to the fourth edition of the World Health Organization classification of skin tumors and the staging was updated to the 8th edition of the Union for International Cancer Control (UICC) TNM Classification of Malignant Tumours [13, 14]. The pathologist was blind to patient’s residency area Patients were treated according to the National Italian Medical Oncology Association (AIOM) guidelines [15].

### Data collection

All data were extracted from a local database. Data collection included demographics (residence at diagnosis, age at diagnosis, sex), tumor characteristics (subtype of melanoma, primary site, Breslow thickness, ulceration, number of mitoses, pTNM stage) and follow-up information. Follow-up data were extracted from scheduled visits. Overall survival (OS) was calculated from date of diagnosis to date of death or last visit. Disease-specific survival (DSS) was calculated from date of diagnosis to date of disease-related death, or date of last visit/disease-unrelated death. Disease-free survival (DFS) was calculated from date of diagnosis to date of recurrence, or date of last visit/death. Recurrence could include local recurrence, regional skin/in-transit metastases, regional lymph node metastases and/or distant metastases.

### Statistical analysis

Continuous data were summarized as median and interquartile range (IQR). Comparisons between exposed and unexposed cohorts were performed using the Chi Square test or Fisher’s exact test (categorical data), and Mann–Whitney test (continuous data). A logistic regression model was estimated to assess the relationship between PFAS exposure and presence of mitoses, adjusting for age and sex. Survival estimates were calculated using the Kaplan–Meier method and compared between exposed and unexposed cohorts using log-rank test (unadjusted analysis) and Cox regression models with unbalanced baseline characteristics as additional independent variables (adjusted analysis). Effect sizes were reported as odds ratio (OR) or hazard ratio (HR) with 95% confidence interval (CI). No adjustment for multiple testing was applied given the exploratory purpose of the study. All tests were two-sided and a p-value of less than 0.05 was considered statistically significant. Statistical analyses were performed using R 4.1 (R Foundation for Statistical Computing, Vienna, Austria) [16].

## Ethics considerations

The study was approved by the Ethics Committee of the Veneto Institute of Oncology (CESC-IOV) on 20 January 2020 (Approval n° 2/2020). The study was conducted according to Helsinki Declaration principles, and all patients gave their consent to have their anonymized data used for scientific purposes.

## Results

### Patients

The analysis included 194 melanoma patients living in the PFAS areas (“exposed cohort) and 488 melanoma patients living outside the PFAS areas (“unexposed cohort). All patients were Caucasian and aged > 15 years. Demographics and tumor characteristics are reported in Table 1. The presence of mitoses was higher in exposed vs. unexposed patients (70.5% vs. 58.7%, *p* = 0.005; Table1), and the association was confirmed when adjusting for age and sex (OR 1.68, 95% CI 1.17 to 2.43; *p* = 0.005). No other statistically significant differences were found in the two cohorts (Table 1).

**Table 1:** Demographics and tumor characteristics according to the geographical area of residency

	Exposed cohort (*n* = 194)	Unexposed cohort (*n* = 488)	*p*-value
Age, years ^a^	50 (37–62)	49 (38–61)	0.90
Males	96 (49.5)	227 (46.5)	0.54
Primary site:			
Acral	12 (6.2)	28 (5.7)	0.93
Head/neck	18 (9.3)	40 (8.2)	
Upper limb	43 (22.2)	120 (24.6)	
Trunk	92 (47.4)	235 (48.2)	
Lower limb	29 (14.9)	65 (13.3)	
Breslow thickness, mm ^ab^	0.9 (0.5–2.0)	0.8 (0.4–1.8)	0.23
Ulceration: ^c^			
Absent	144 (76.6)	372 (79.3)	0.51
Present	44 (23.4)	97 (20.7)	
Presence of Mitoses ^d^	134 (70.5)	284 (58.7)	**0.005**
pTNM:			
I	116 (59.8)	325 (66.6)	0.22
II	43 (22.2)	85 (17.4)	
III	35 (18.0)	78 /16.0)	
Subtype: ^e^			
ALM	4 (2.0)	14 (3.0)	0.78
LMM	5 (2.7)	7 (1.5)	
NM	33 (17.6)	85 (18.1)	
SSM	141 (75.0)	347 (73.8)	
Other ^f^	5 (2.7)	17 (3.6)	

Data expressed as n (%) or a median (IQR). Data not available in b32, c25, d8 and e24 patients. fOther subtypes included desmoplastic (4 patients), neurotropic (1 patient), nevoid (8 patients), pagetoid (1 patient), polypoid (2 patients) and spitzoid (6 patients). *ALM* Acral Lentiginous Melanoma, *LMM* Lentigo Maligna Melanoma, *NM* Nodular Melanoma, *SSM* Superficial Spreading Melanoma

### Survival

**Fig. 2:**
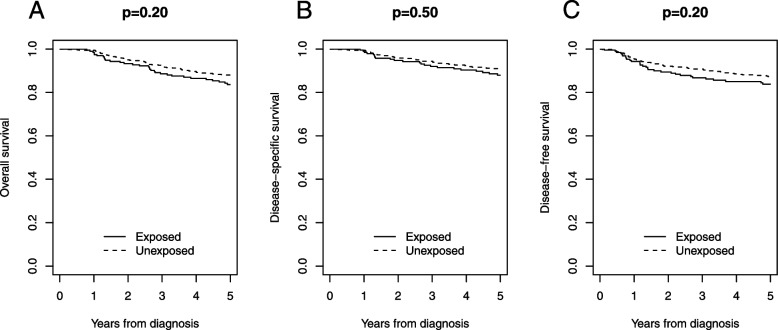
Overall survival **A**, disease-specific survival **B**, and disease-free survival **C**: comparison between exposed and unexposed cohorts

At a median follow-up of 90 months (IQR 59–136), overall 135 patients died (84 from the disease and 51 due to other causes) and 542 were alive, while the information was not available in five patients who were lost to follow-up. 5-year OS was 83.7% in exposed patients and 88.0% in unexposed patients (*p* = 0.20); 5-year DSS was 88.0% in exposed patients and 90.9% in unexposed patients (*p* = 0.50); 5-year DFS was 83.8% in exposed patients and 87.3% in unexposed patients (*p* = 0.20) (Fig. 2). Adjusting for imbalanced characteristics at baseline (presence of mitoses), survival was not statistically different between exposed and unexposed patients (OS: HR 1.10, 95% CI 0.77 to 1.58, *p* = 0.57; DSS: HR 0.99, 95% CI 0.62 to 1.59, *p* = 0.99; DFS: HR 1.10, 95% CI 0.74 to 1.64, *p* = 0.62), while higher number of mitoses per mm2 was associated with impaired OS (HR 5.87, 95% CI 3.24 to 10.65; *p* < 0.0001), DSS (HR 7.85, 95% CI 3.41 to 18.05; *p* < 0.0001) and DFS (HR 7.05, 95% CI 3.55 to 13.95; *p* < 0.0001).

## Conclusions

In the last decade, the contamination of water by PFAS has gained increasing interest in medical research. Previous studies showed an association between PFAS exposure and testicular Leydig cell adenomas, pancreatic and hepatocellular carcinoma or thyroid tumor in the rats [8, 17, 18], as well as neonatal mortality, neurotoxicity and metabolic alterations [19]. Many recent studies investigated the serum PFAS concentration and reported associations with cardiovascular disease, reproductive disorders, Alzheimer’s or some neoplasms [20–25]. Available scientific literature offers limited information on the role of PFAS in melanoma development/progression. A recent scoping review found sparse evidence on the relationship between PFAS and melanoma in epidemiologic studies [8]. On the other hand, a previous laboratory study on developing rats found that exposure to perfluorooctane sulfonate (PFOS) potentially altered pathways associated with different cancers including melanoma [26]. Our findings suggest that exposure to PFAS was associated with higher presence of mitoses in human melanoma, while other tumor characteristics (e.g., site, Breslow thickness, ulceration, stage, and subtype) were comparable between exposed and unexposed cohorts. Previous studies suggested that PFAS exposure may cause alterations in telomere length and vitamin D metabolism [27–29], with a potential cascade on cancer pathogenesis. In 2015, a systematic review showed that cutaneous melanoma was associated with telomere lenghtening, although the underlying mechanisms is not fully understood yet [30]. The endocrine disrupting action of perfluoro-octanoic acid (PFOA) is one of the predominant forms in human samples of PFAS. Due to the molecular similarity between vitamin D and steroid hormones, PFOA competes with 1,25-dihydroxyvitamin D on the same binding site in its receptor, which is involved in the genomic actions of vitamin D (cell cycle progression, differentiation and apoptosis, immunomodulation) [29]. The binding with PFAS could be responsible for the deficiency or the inactivation of vitamin D, thus altering those signaling pathways [31]. Literature suggests that vitamin D and its derivates may have anti melanoma development properties, promoting cellular differentiation and inhibiting proliferation [11, 32–34]. Previous studies reported an inverse association between vitamin D levels and number of mitoses [32–35], which may contribute to explain the higher number of mitoses in PFAS-exposed cohort in our study. In our series, mitotic index was strongly associated with patient survival as reported by many other investigators [36–38]. However, our data did not suggest an association between PFAS exposure and patient survival. We acknowledge that such relationship is complex and may be found in subgroups of patients which had small size in our analysis. Therefore, we cannot exclude that PFAS exposure may have some impact on patient survival, or provide indications about the clinically significance of such impact. Moreover, the heterogeneity in terms of source of water within the exposed cohort might have influenced the magnitude of PFAS exposure. Of note, our study has some limitations that should be considered by the reader. First, the retrospective design limited data availability. For example, plasma level of PFAS at diagnosis was not available, and patients were stratified according to area of residency and IRSA-CNR analysis on drinking waters. Second, the majority of patients were diagnosed with a stage I melanoma and a 10-y OS will be more appropriate for this stage. Hence, we cannot exclude that actual PFAS contamination may have a prognostic role in melanoma patients. Third, the generalizability of the findings may be limited to similar settings. Finally, no adjustment for multiple testing was applied (because of the exploratory purpose of the study) hence further studies are required to confirm the findings. In conclusions, our data showed that melanoma patients living in PFAS-contaminated areas (thus potentially exposed to PFAS) had higher presence of mitoses compared to unexposed patients, but this association did not translate into a survival difference. However, the limitations of the study (mainly regarding the unquantifiable magnitude of PFAS exposure in our patients) suggest caution when drawing any conclusions, hence further studies may provide a better understanding of the impact of PFAS exposure on the biological aggressiveness of cutaneous melanoma. 

## Data Availability

The datasets presented in this study can be found in online repositories. The names of the repository/repositories and accession number(s) can be found below: 10.5281/zenodo.6976964 (accessed on 2 May 2022).

## Abbreviations

PFAS: Per- and polyfluoroalkyl substances
PFOA: Perfluoro-octanoic acid
PTFE: Polytetrafluoroethylene
IRSA-CNR: Italian National Research Center
OS: Overall survival
DSS: Disease-specific survival
DFS: Disease-free survival
UICC: Union for International Cancer Control
AIOM: Italian Medical Oncology Association
IQR: Interquartile range
CI: Confidence interval
CESC-IOV: Ethics Committee of the Veneto Institute of Oncology
ALM: Acral Lentiginous Melanoma
LMM: Lentigo Maligna Melanoma
NM: Nodular Melanoma
SSM: Superficial Spreading Melanoma
